# Oleanolic Acid Reduces Hyperglycemia beyond Treatment Period with Akt/FoxO1-Induced Suppression of Hepatic Gluconeogenesis in Type-2 Diabetic Mice

**DOI:** 10.1371/journal.pone.0042115

**Published:** 2012-07-30

**Authors:** Xiao-Yi Zeng, Yi-Ping Wang, James Cantley, Tristan J. Iseli, Juan Carlos Molero, Bronwyn D. Hegarty, Edward W. Kraegen, Yang Ye, Ji-Ming Ye

**Affiliations:** 1 Molecular Pharmacology for Diabetes, Health Innovations Research Institute and School of Health Sciences, RMIT University, Melbourne, Victoria, Australia; 2 Diabetes and Obesity Program, Garvan Institute of Medical Research, Sydney, New South Wales, Australia; 3 Shanghai Institute of Materia Medica, Chinese Academy of Sciences, Shanghai, China; The University of Hong Kong, Hong Kong

## Abstract

The present study investigated the chronic efficacy of oleanolic acid (OA), a triterpenoid selected from our recent screening, on hyperglycemia in type-2 diabetic mice. C57BL/6J mice were fed a high-fat diet followed by low doses of streptozotocin to generate a type-2 diabetic model. OA (100 mg/kg/day) was administered orally for 2 weeks with its effects monitored for 6 weeks. High-fat feeding and streptozotocin generated a steady hyperglycemia (21.2±1.1 mM) but OA administration reversed the hyperglycemia by ∼60%. Interestingly, after the cessation of OA administration, the reversed hyperglycemia was sustained for the entire post-treatment period of the study (4 weeks) despite the reoccurrence of dyslipidemia. Examination of insulin secretion and pancreas morphology did not indicate improved β-cell function as a likely mechanism. Urine glucose loss was decreased with substantial improvement of diabetic nephropathy after the OA treatment. Pair-feeding the OA-treated mice to an untreated group ruled out food intake as a main factor attributable for this sustained reduction in hyperglycemia. Studies with the use of glucose tracers revealed no increase in glucose influx into muscle, adipose tissue or liver in the OA-treated mice. Finally, we analyzed key regulators of gluconeogenesis in the liver and found significant increases in the phosphorylation of both Akt and FoxO1 after treatment with OA. Importantly, these increases were significantly correlated with a down-regulation of glucose-6-phosphatase expression. Our findings suggest triterpenoids are a potential source of new efficacious drugs for sustained control of hyperglycemia. The liver appears to be a major site of action, possibly by the suppression of hepatic glucose production via the Akt/FoxO1 axis.

## Introduction

The incidence of diabetes is estimated at 220 million worldwide [1] and prolonged hyperglycemia is a major cause of various diabetic complications including nephropathy [2]. Effective control of blood glucose is, therefore, crucial to the treatment of diabetes and the prevention/delay of diabetic complications. Type-2 diabetes accounts for ∼90% of all diabetes cases [1] and it results from the metabolic disorders of insulin resistance (diminished sensitivity of the target tissues to insulin action) and β-cell failure (reduced ability of the pancreatic β-cells to produce sufficient insulin). Therefore, improvements of insulin action and β-cell function are important mechanisms for the pharmacological treatment of type-2 diabetes.

Sustained control of hyperglycemia is of great importance to the treatment of type-2 diabetes and it remains a significant challenge. Until recently, the mainstay oral medications to improve insulin action in type-2 diabetes have been biguanides (e.g.metformin) and thiazolidinediones (TZDs) [3]. However, biguanides are not adequate therapies on their own in the long-term [3], [4] as they have limited effects in improving insulin action in muscle [5], [6]. While TZDs are effective in lowering hyperglycemia, largely by an insulin sensitizing action [7], [8], concerns over the adverse effects of TZDs on an increased risk of heart failure [9] and bladder cancer [10] have restricted their long-term use. Other new drugs such as GLP analogues and inhibitors of the sodium glucose co-transporter (SGLT) appear promising [11], [12], [13], however their long term effectiveness is not clear. Thus there remains an urgent need for the development of new anti-diabetic drugs with sustained efficacy.

We recently found that triterpenoid compounds isolated from bitter melon have potent efficacy in stimulating GLUT4 translocation in L6 myotubes and 3T3L1 adipocytes, along with activation of the AMPK pathway [14] Our acute studies in mice showed that triterpenoids are able to reduce glucose intolerance in insulin resistant high-fat (HF)-fed mice after a single injection [14]. These findings are encouraging because triterpenoids are a rich natural source for drug discovery, with more than 20,000 of them known to exist in plants [15]. The present study investigated whether the triterpenoid, oleanolic acid (OA), is an effective treatment for hyperglycemia in a murine model of type-2 diabetes. The study focused on the OA compound based on our recent screens [14].

OA itself has been used in humans for its potential therapeutic application for cancer [15] and an OA analogue has been shown to alleviate diabetic nephropathy in type-2 diabetic patients [16]. OA and its analogues have been shown to lower hyperglycemia in STZ-treated rodents [17], HF-fed or *db/db* mice [18], to protect against diabetic nephropathy [17] and to enhance the survival of pancreatic islets [19]. However, all of these studies were relatively short-term (mostly less than 2 weeks) and the sustainability of these therapeutic effects is not known. Here we investigated the therapeutic efficacy of OA in reducing hyperglycemia in a murine diabetic model produced by chronic HF feeding combined with low doses of STZ [20], [21]. We demonstrate a potent glucose-lowering effect that was sustained well beyond the treatment period and was accompanied by a significant improvement in renal structures. Furthermore, we have identified Akt/FoxO1 mediated suppression of glucose-6-phosphatase (G6Pase), a key regulator of hepatic glucose production, as a likely mechanism underlying the improved glucose homeostasis.

## Materials and Methods

### Animal model

Male C57BL/6J mice (10 weeks old) were purchased from the Animal Resources Centre (Perth, Australia). The animals were kept in a temperature-controlled room (22±1°C) on a 12-h light/dark cycle with free access to food and water. After 1 week of acclimatization, mice were fed *ad libitum* for 10 weeks with a standard lab chow diet (CH; 8% calories from fat, 21% calories from protein, and 71% calories from carbohydrate) or a high-fat diet (HF; 45% calories from fat, 20% calories from protein, and 35% calories from carbohydrate) in order to induce insulin resistance [7], [8], [14], [22]. Mice were then injected with either vehicle (saline) or a low dose of streptosotocin (STZ, 40 mg/kg/day) for five consecutive days in order to induce diabetes (fasting blood glucose >12 mM) [20], [21]. CH-fed mice treated with STZ comprised a model of type-1 diabetes (T1D mice). HF-fed mice treated with STZ comprised a model of type-2 diabetes (T2D mice). One week after the last STZ injection (‘baseline’), a subset of T1D and T2D mice received OA as a food additive at 100 mg·kg^−1^·day^−1^ for two weeks (T1D-OA and T2D-OA respectively). This dose of OA was selected on the basis of our previous study [14]. The remaining T1D and T2D mice received their normal CH or HF diet (T1D-Veh and T2D-Veh, respectively). Body weight, food intake and fasting blood glucose levels were monitored on a weekly basis until 4 weeks after the cessation of OA administration. All experiments were approved by the Animal Ethics Committees of the Garvan Institute (#0847) and RMIT University (#1012) in accordance with the guidelines of the National Health and Medical Research Council of Australia.

### Assessment of effects on hyperglycemia, blood insulin level and glucose tolerance

Blood glucose levels were measured once a week after 5–7 hours of fasting. Blood samples were collected from the tail tip and were analyzed using a glucometer (AccuCheck II; Roche, New South Wales, Castle Hill, Australia). Two weeks after the cessation of OA treatment, i.p. glucose tolerance tests (ipGTT; glucose load 1 g/kg BW) were performed in the 5–7 hours fasted state, as previously described [22]. Briefly, blood samples were obtained from the tail tip at 0, 15, 30 and 60 min for the measurement of blood glucose levels and at 0, 5, 30 and 60 min for the measurement of insulinemia (determined by radioimmunoassay; Linco/Millipore, Billerica, MA).

### Insulin secretion assays, pancreatic histology and insulin content measurements

Islet isolation and *ex vivo* insulin secretion assays were performed as previously described [23]. Mice were killed by cervical dislocation and the pancreas was perfused with 2 ml of Liberase (Roche, Basel, Switzerland) solution (0.25 mg/ml in Krebs-Ringer buffer) via injection into the common bile duct. After pancreatic digestion at 37°C, islets were purified using a Ficoll-paque (GE Healthcare, Chalfont St. Giles, U.K.) gradient. Islets were washed and immediately pre-incubated for 1 hr in Krebs-Ringer buffer containing HEPES (KRBH), 0.1% BSA and 2 mM glucose. Batches of five islets were incubated at 37°C for 1 h in 130 µl KRBH containing 0.1% BSA and 2, 5.5, 11 or 20 mM glucose. For the measurement of pancreatic insulin content, the pancreas was weighed and then homogenized in ice-cold acid ethanol (0.15 M HCl in 75% ethanol) immediately after collection. Insulin concentrations in the incubation medium and pancreatic extracts were determined by a commercial insulin radioimmunoassay kit (Linco/Millipore, Billerica, MA).

Quantification of β-cell area was performed based on previous methods [24]. Each pancreas was removed, cleared of fat and lymph nodes, fixed in 10% neutral buffered formalin and embedded in paraffin wax. 5 µm sections were cut and incubated for 30 min at room temperature with blocking solution (PBS buffer with 2% BSA and 5% chick serum) before incubation overnight at 4°C in blocking solution containing mouse anti-insulin antibody (I2018, Sigma-Aldrich). Sections were then incubated with chicken anti-mouse IgG-Alexa Fluor 594 conjugate (Invitrogen) for 1 hr at room temperature in darkness. Transmitted light images were captured (magnification ×20). To quantitate β-cell area, the outline of the pancreas section and all insulin-positive cells were traced and scored using ImageJ image analysis software (ImageJ, NIH, Bethesda, MD). Results are expressed as the percentage of the total pancreatic area stained positive for insulin.

### Assessment of the effects on urine glucose secretion and kidney morphology

Urine samples were collected in the morning three weeks after the completion of OA treatment. The urinary glucose level was measured by the glucose oxidase assay using an automated glucose analyser (YSI 2300 Stat Plus, Yellow Springs Instruments, Yellow Springs, Ohio, USA). For the kidney morphology study, kidneys were rapidly removed after cervical dislocation. Coronal sections of renal tissue were immersion-fixed in 10% neutral buffered formalin and embedded in paraffin. Sections 5 µm thick were stained with periodic acid-Schiff and evaluated using methods described previously [25]. To quantitate tubular atrophy, the tubule cell height of an individual cortical tubule was measured using line morphometry (magnification ×200) by ImageJ. A total of 50 randomly-selected cortical tubules in 10 non-overlapping fields (magnification ×200) were measured, and the mean cross-sectional tubule cell height was determined for each section. The degree of glomerular hypertrophy was measured quantitatively. The outline of the glomeruli and glomerular capillary tuft was traced, and the computed area was used as a measure of total glomerular area and tuft area. The mean value of 20 randomly selected glomeruli was determined for each section. The cortical interstitial volume included the tubular basement membrane and peritubular capillaries. To quantitate this area, cortical fields (magnification ×200) were viewed on a video screen, and the area of interstitial space was determined with image analysis software and expressed as a percentage of the total area of the field. The mean percentage area of five non-overlapping cortical fields was calculated for each section.

### Measurement of plasma and liver triglyceride

Plasma was extracted from blood samples collected from the tail tip and stored at −80°C. Mice were killed by cervical dislocation in the fasted state and liver samples were immediately freeze-clamped. Liver triglycerides were extracted by the method of Folch. The triglyceride level in plasma and liver extract was determined by a Peridochrom triglyceride GPO-PAP kit (Roche Diagnostics) as previously described [7], [8], [12], [22].

### Measurement of glucose flux in key tissues for glucose homeostasis

In one subset of mice, [^3^H] labeled 2-deoxy-D-glucose (2DG; PerkinElmer, USA) and D-[^14^C] glucose (PerkinElmer, USA.) were used to measure glucose metabolism in skeletal muscle, fat and liver, as described in our previous work [8]. Briefly, 4 weeks after the cessation of OA treatment, an ipGTT was performed after 5–7 hours of fasting. Glucose (1 g/kg BW) containing [^3^H]-2DG (65 µCi/kg BW) and D-[^14^C] glucose (32 µCi/kg BW) was injected *ip*. Plasma samples were obtained from the tail tip at 10, 20, 30 and 40 min after glucose administration for estimation of plasma tracer concentration. At the completion of the ipGTT, mice were culled and tissue samples were immediately freeze-clamped for subsequent analysis.

### Analysis of gene expression

Total RNA was extracted from liver tissue using TRIZOL® (Invitrogen, USA) according to the manufacturer's instructions. Reverse transcription was carried out with 0.2 mg of RNA using the High Capacity cDNA Reverse Transcription Kit (Applied Biosystems, USA). Real time PCR was carried out using the IQ SYBR Green Supermix (2×) (Bio-Rad Laboratories Inc, USA) for G6Pase and PEPCK (Genework, Australia). The gene expression from each sample was analyzed in duplicate and normalized against the housekeeper, 18S. The primer sequence (5′ to 3′) of 18S was: CGCCGCTAGAGGTGAAATTCT (sense) and CGAACCTCCGACTTTCGTTCT (antisense); PEPCK: CCACAGCTGCTGCAGAACA (sense) and GAAGGGTCGCATGGCAAA (antisense); G6Pase: AACGCCTTCTATGTCCT CTTTC (sense) and GTTGCTGTAGTAGTCGGTGTCC (antisense). All reactions were performed on the iQ™ 5 Real Time PCR Detection System (Bio-Rad Laboratories Inc, USA). The results are expressed as relative gene expression using the ΔCt method.

### Western blotting

Western blotting in liver samples was performed as described previously [26] with minor modifications. Briefly, the freeze-clamped liver tissues were homogenized in ice-cold lysis buffer at pH 7.5 containing (in mM): 50 Tris, 150 NaCl, 1% Triton X-100, 10 NaP, 100 NaF, 2 Na3VO4, 1 EDTA, 1 EGTA and 10% glycerol supplemented with protease inhibitor cocktail tablets (Roche Diagnostics Pty Ltd, Australia) and DL-dithiothreitol. Total protein concentrations were assessed using Bio-Rad Protein Assay (Bio-Rad Laboratories, Hercules, CA) before protein samples were denatured in SDS sample buffer (125 mM Tris-HCl, pH 6.8, 50% glycerol, 2% SDS, 5% β-mercaptoethanol and 0.01% bromophenol blue). Regulation of glucoenogenesis was assessed by total- and phospho(Ser473)-Akt, as well as total- and phospho(Ser256)-Forkhead box protein O1 (FoxO1, Cell Signaling, USA). Densitometry analysis was performed using Image Lab software (Bio-Rad Laboratories, USA) and representative blots are shown.

### Statistical Analyses

Data are presented as means ± SE. One-way analysis of variance was used for comparison of relevant groups. When significant variations were found, the Tukey-Kramer multiple comparisons test was applied. Pearson's two-sided correlation was used for the correlation analysis. Differences at *p*<0.05 were considered to be statistically significant.

## Results

### Induction of type-1 and type-2 diabetes

We first induced a type 2 diabetes model of hyperglycemia by chronic HF-feeding and low-dose STZ based on previous reports [20], [21]. As shown in Table 1, HF feeding caused a small increase in body weight and a 2.3 fold increase in epididymal fat compared to CH mice. HF mice also exhibited increased blood insulin (∼40%) and liver triglyceride (2.4 fold) compared to CH mice, but the circulating levels of glucose and triglyceride were similar between HF and CH mice. CH-fed mice treated with STZ (T1D thereafter), demonstrated moderate increases in blood glucose and plasma triglyceride compared to CH mice (∼50–60%, p<0.05 vs. CH-fed mice), but liver triglyceride content was unchanged. As expected, HF-STZ (T2D thereafter) mice recapitulated several major characteristic metabolic disorders in type-2 diabetes, namely hyperglycemia (∼2 fold), hypoinsulinemia (by 20%), dyslipidemia (80%) and liver steatosis (2.5 fold) compared to CH mice. Consistent with previous reports [21], T2D mice also displayed a lack of glucose response to insulin action during an insulin tolerance test - a typical feature of insulin resistance (data not shown). Compared with HF feeding alone, T2D mice displayed hyperglycemia (>2 fold, p<0.01), hypertriglyceridemia (50%, p<0.05) and reduced plasma insulin level (50%, p<0.01) while retaining a similar level of liver steatosis. Compared with T1D, T2D mice showed more epididymal fat (30%), greater hyperglycemia (∼30%) and significantly increased triglyceride content in the liver (2 fold).

**Table 1:** Metabolic characteristics of the type-1 and type-2 diabetes mouse models.

	CH (Normal)	HF (IR)	CH-STZ (T1D)	HF-STZ (T2D)
Body weight (g)	29.5±0.4	31.7±0.8†	28.8±0.4†	27.8±0.2† ** #
Epi fat (% BW)	1.4±0.1	3.2±0.3††	1.2±0.1†	1.6±0.1** #
Blood glucose (mM)	9.2±0.2	8.3±0.4	13.6±0.6††	17.5±1.2†† ** #
Blood insulin (pg/ml)	367±29	503±130†	300±26	301±63† *
Plasma triglyceride (mM)	1.2±0.1	1.5±0.1	1.9±0.1††	2.2±0.2**
Liver triglyceride (µmol/g)	7.8±0.8	18.6±2.0††	9.4±0.9	19.6±1.1†† ** ##

C57BL/6J mice were fed with chow diet and injected with saline (CH) or STZ (T1D), or fed with HF diet and injected with saline (HF) or STZ (T2D). IR, insulin resistant. Blood samples were collected from mice in the 5–6 hour fasted state. Blood glucose (n = 8–10) and insulin (n≥5), and plasma triglyceride (n = 7–8) were measured as described in the methods. Body weight, epididymal weight and liver triglycerides were measured at the end of the study (n≥10). Data are expressed as means ± SE.

† p<0.05,

†† p<0.01 vs. CH;

* p<0.05,

** p<0.01 vs. HF;

# p<0.05,

## p<0.01 vs. T1D.

### Effects of OA on hyperglycemia and glucose tolerance

**Figure 1:**
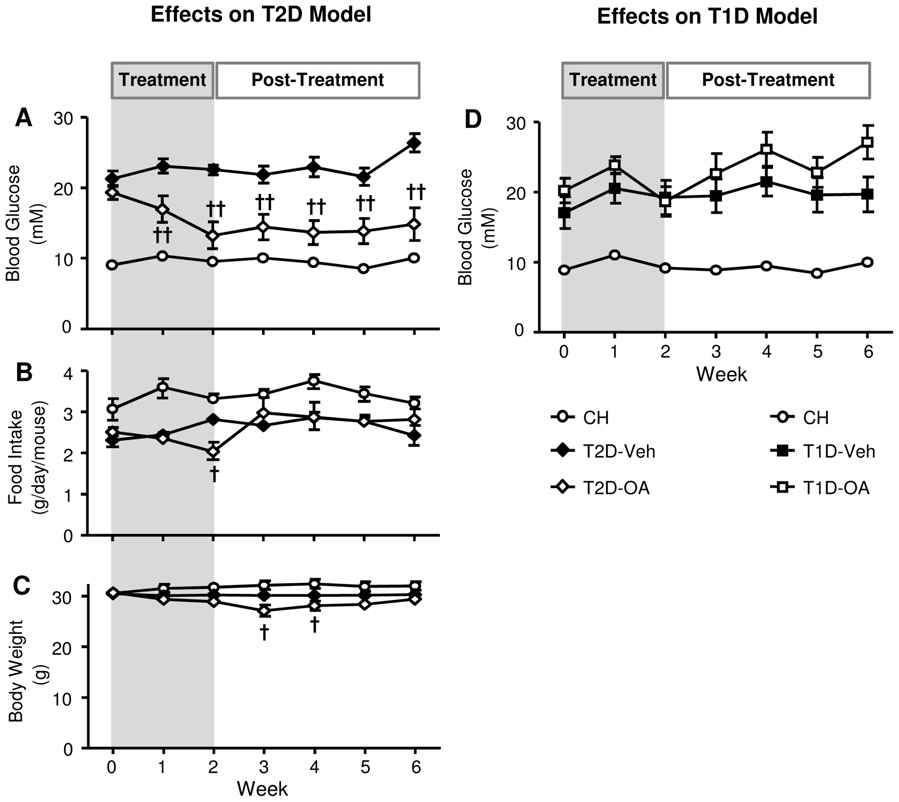
Effects of OA on blood glucose, food intake and body weight in T2D and T1D mice over time. HF-fed mice with STZ injections were treated with (T2D-OA) or without (T2D-Veh) OA in the diet for two weeks, at the end of which OA was removed from the diet. Blood glucose, food intake and body weight were monitored between 14:00 and 16:00 once a week (*A, B* and *C*). CH-fed mice with STZ injections were treated with (T1D-OA) or without (T1D-Veh) OA in the diet for two weeks, at the end of which OA was removed from the diet. Effects of OA on hyperglycemia in T1D mice (D). CH, normal chow fed mice. Data are expressed as means ± SE. † p<0.05, †† p<0.01 vs. T2D-Veh group, n = 11–16 per group.

Oral administration of OA, at a dose of 100 mg/kg/day, dramatically reversed the hyperglycemia of T2D mice by more than 60% (approaching the level of CH-fed mice) by the end of the two-week treatment (22.5±0.6 vs. 13.2±1.9 mM, p<0.001). Intriguingly, following the removal of OA from the HF diet, the reversed hyperglycemia evident in the T2D-OA group persisted for the rest of the study. Meanwhile the hyperglycemia in the untreated T2D-Veh group remained high (Fig. 1*A*). Under a similar treatment regime, OA showed no significant effect in lowering the hyperglycemia evident in the T1D Figmouse model (Fig. 1*D*). Along with a reduced food intake at the end of the two-week treatment (Fig. 1*B*), T2D-OA group had a lower body weight than T2D-Veh group at week 3 and 4 (Fig. 1*C*). However, food intake and body weight of T2D-OA mice was completely recovered by the end of the study (Fig. 1, *B* and *C*).

**Figure 2:**
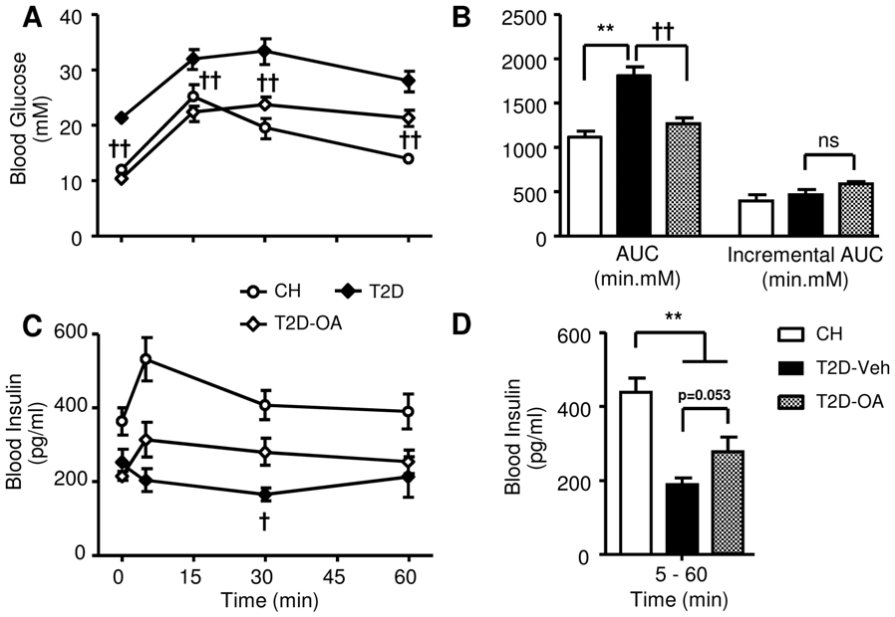
Effects of OA on glucose tolerance and blood insulin. Studies were performed in mice two weeks after the removal of OA. ipGTT was performed with an injection of glucose (1 g/kg, *ip*) after 5–7 hours of fasting. Blood glucose was monitored at 0, 15, 30, and 60 min following the glucose injection (*A*). ipGTT results were quantified by calculating the area under the blood glucose curve (AUC) and the incremental AUC (iAUC) (*B*). Insulin levels throughout the ipGTT (*C*). The average value of blood insulin levels from 5 to 60 mins during the ipGTT (*D*). CH, normal chow fed mice; T2D-Veh, HF-fed mice with STZ injections; T2D-OA, HF-fed mice with STZ injections and OA treatment. ** p<0.01 vs. CH; †† p<0.01 vs. T2D-Veh, n = 5–8 per group.

As the anti-hyperglycemic effect of OA was only observed in T2D mice, our subsequent studies were performed only in the T2D mouse model. OA significantly improved the glucose tolerance of T2D mice as evidenced by decreased blood glucose level in T2D-OA compared to T2D-Veh at all time points (p<0.01) during the ipGTT as well as by a reduced area under the curve (Fig. 2, *A* and *B*). However, when expressed as the incremental area under the curve, we did not find a significant difference between the T2D-Veh and T2D-OA groups. During the ipGTT, blood insulin levels were significantly higher in T2D-OA compared to T2D-Veh mice at 30 min, with a similar trend at 5 min (p = 0.082) (Fig. 2*C*). However, when expressed as the average value from 5 to 60 min, there was no statistical difference between the T2D-Veh and T2D-OA groups (p = 0.053, Fig. 2*D*).

### Insulin secretion, β-cell numbers and pancreatic insulin content post-OA treatment

**Figure 3:**
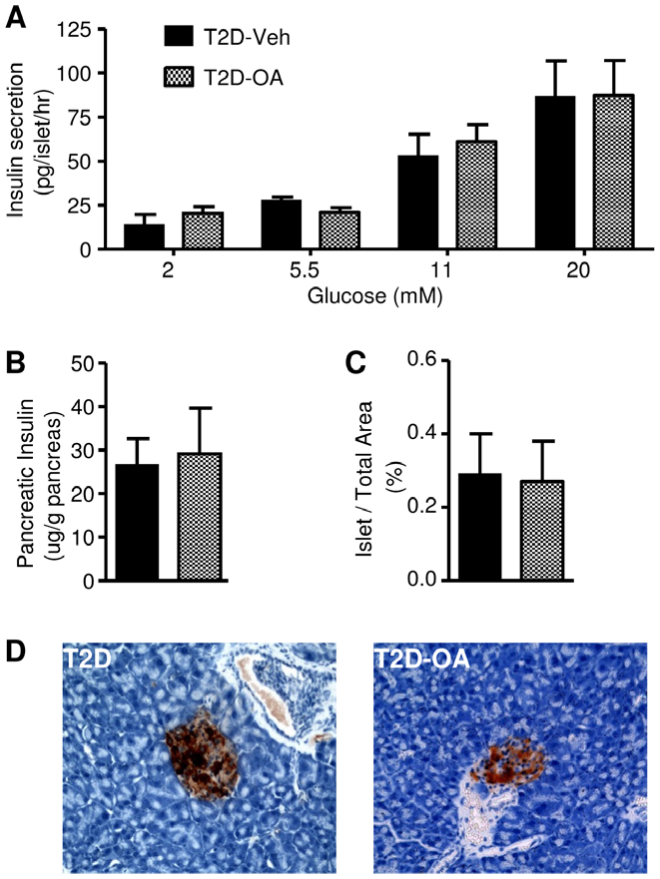
Effects of OA on insulin secretion and insulin in pancreatic β-cells. Four weeks after the cessation of OA treatment, fresh islets were isolated from T2D mice treated with or without OA, and insulin secretion in response to different glucose concentrations was measured (*A*). Pancreatic insulin content (*B*). β-cell area (expressed as a percentage of pancreatic area) (*C*). Representative images of immunohistochemical staining of β-cells (*D*). T2D, HF-fed mice with STZ injections; T2D-OA, HF-fed mice with STZ injections and OA treatment. n = 4–6 per group.

Fig. 2*C* and *D* appeared to indicate a seeming improvement in β-cell function after treatment of T2D mice with OA, which could provide a mechanism for the improved blood glucose levels in these mice. We therefore further assessed glucose-stimulated insulin secretion in isolated islets. As shown in Fig. 3*A*, islets isolated from T2D-OA mice showed an insulin secretion response to increasing glucose stimulation that was indistinguishable from T2D-Veh mice. We next examined total pancreatic insulin level and found that the administration of OA had no effect on the total pancreatic insulin content in T2D mice (29.1±10.6 vs. 26.6±6.0 µg/g pancreas in the T2D-Veh group, p>0.05) (Fig. 3*B*). Finally, immunohistochemical staining of pancreatic sections showed that OA did not affect the total number of β-cells per pancreas in T2D mice (0.29±0.11% vs. 0.27±0.11%, p = 0.894) (Fig. 3*C and D*).

### Alleviation of glucose loss in urine and nephropathy post-OA treatment

**Figure 4:**
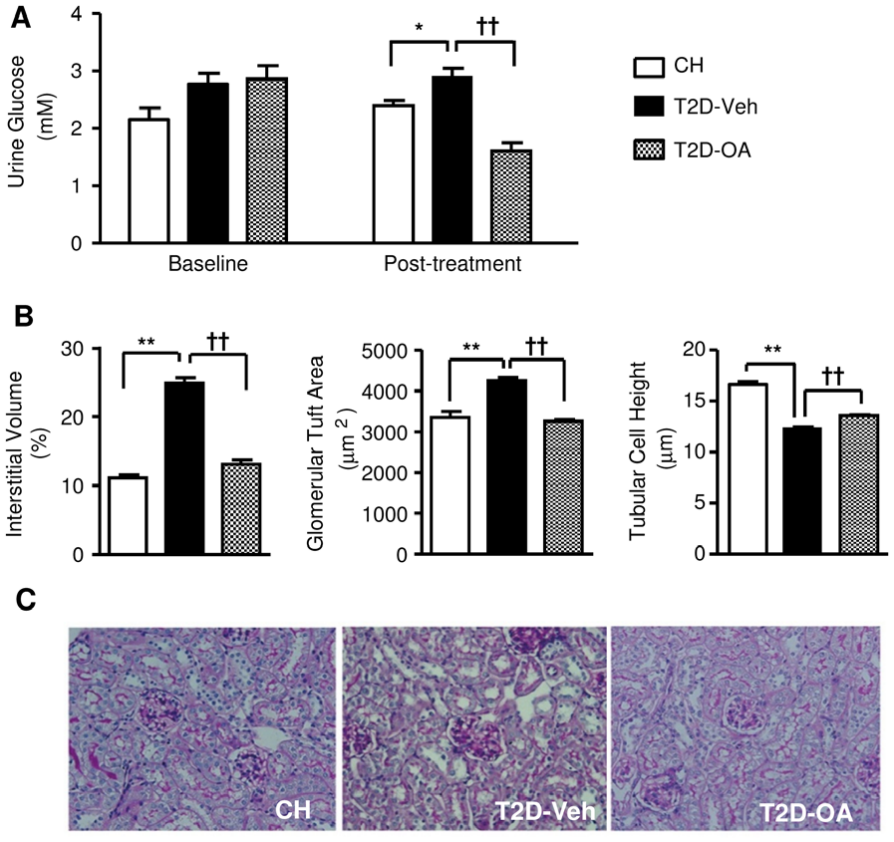
Effects of OA on glucose levels in urine and kidney morphology. Urine glucose levels in CH, T2D-Veh and T2D-OA mice were measured before the OA treatment began and at the end of the study (4 weeks after cessation of OA treatment) (*A*). Kidneys were harvested and coronal sections of renal tissues were stained with periodic acid-Schiff for the quantification of interstitial volume, glomerular tuft area and tubular cell height (*B*). Representative images of stained kidney sections (*C*). T2D-Veh, HF-fed mice with STZ injections; T2D-OA, HF-fed mice with STZ injections and OA treatment. * p<0.05, ** p<0.01 vs. CH; †† p<0.01 vs. T2D-Veh, n = 5–10 per group.

In order to investigate if the reduced blood glucose level in T2D-OA mice was due to an increase in the urine excretion of glucose, we measured the glucose level in the urine of T2D mice before and after the treatment with OA. TZD mice excreted more glucose in the urine compared to CH-fed mice at baseline (Fig. 4*A*). Whilst glucose excretion remained high in T2D mice when measured 3 weeks after the cessation of OA treatment, TZD-OA mice demonstrated a significant drop in urine glucose levels (11.8±1.2 vs. 5.1±0.8 mM before vs. after OA treatment, p<0.01) (Fig. 4*A*). Morphology studies revealed that the OA treatment dramatically improved kidney structure of T2D mice, as indicated by significant reductions in interstitial volume (13.2±0.7 vs. 24.9±0.8% in untreated T2D, p<0.01) and glomerular turf area in T2D-OA mice (3251±41 vs. 4248±83 um^2^ in untreated T2D-, p<0.01) to levels similar to CH-fed mice (Fig. 4*B,C*). Associated with the normalization of interstitial volume and glomerular turf area, the reduced tubular cell height evident in T2D mice was significantly ameliorated in T2D-OA mice (13.6±0.1 vs. 12.4±0.1 µm in untreated T2D, p<0.01) (Fig. 4*B and C*).

### Triglyceride levels in plasma and liver during and after OA treatment

**Figure 5:**
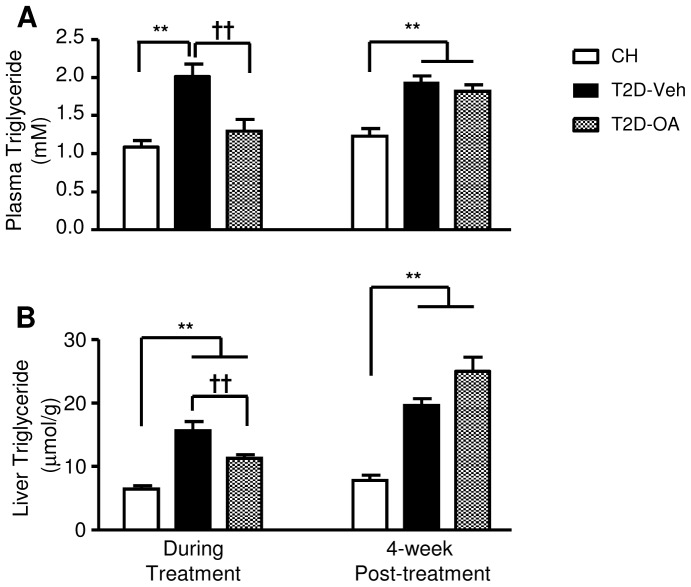
Effects of OA on triglyceride levels in plasma and liver during and after OA treatment. Lipid accumulation in the liver and plasma was assessed both during OA treatment (at week 2) and 4 weeks after the cessation of OA treatment. Mice were euthanized following a 5–7 hour fast and samples of plasma (*A*) and liver (*B*) were collected for the measurement of triglyceride levels. T2D-Veh, HF-fed mice with STZ injections; T2D-OA, HF-fed mice with STZ injections and OA treatment. ** p<0.01 vs. CH; †† p<0.01 vs. T2D-Veh, n = 5–8 per group.

T2D-Veh mice had significantly higher levels of plasma and liver triglyceride compared to CH-fed mice at the end of the two-week treatment period (Fig. 5*A*) and at 4-weeks post-treatment (Fig. 5*B*). As expected, two weeks of OA treatment substantially reduced the triglyceride levels in plasma (Fig. 5*A*) and liver (Fig. 5*B*) of T2D mice. However, 4-weeks after the termination of OA administration, both plasma and liver triglycerides had returned to the levels of the T2D-Veh group (Fig. 5*A, B*).

### Influence of pair-feeding on hyperglycemia and glucose tolerance

**Figure 6:**
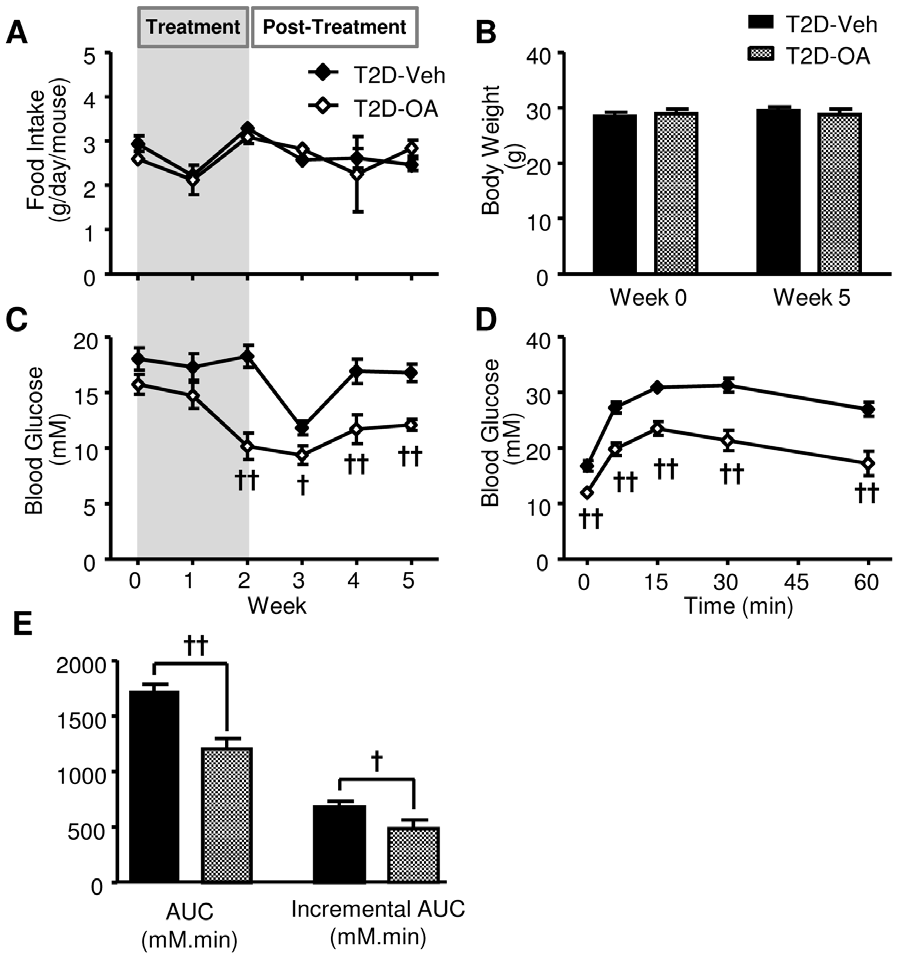
Comparisons of blood glucose and ipGTT between pair-fed T2D-Veh and T2D-OA mice. Food intake (*A*), body weight (*B*) and basal blood glucose (*C*) were monitored throughout the pair-feeding study. ipGTT was performed in mice two weeks after the cessation of OA treatment, with an injection of glucose (1 g/kg, ip) after 5–7 hours of fasting. Blood glucose was monitored at 0, 15, 30, and 60 min following the glucose injection (*D*). ipGTT results were quantified by calculating the area under the blood glucose curve (AUC) and the incremental AUC (iAUC) (*E*). T2D-Veh, HF-fed mice with STZ injections; T2D-OA, HF-fed mice with STZ injections and OA treatment. † p<0.05, †† p<0.01 vs. T2D-Veh, n = 5–10 per group.

Since a fluctuation in food intake was observed in the T2D-OA group during the initial experiment, we carried out an additional pair-feeding study to determine if the sustained reversal of hyperglycemia during the period of post-OA treatment was due to the difference in food intake. The food intake in pair-fed T2D-Veh and T2D-OA groups (2.7±0.1 vs. 2.5±0.1 g/mouse/day, p>0.05) was well matched (Fig. 6*A*). T2D-Veh and T2D-OA groups were not different in body weight at the baseline nor at the end of the study (Fig. 6*B*). The basal blood glucose of the T2D-OA group showed a significant decrease compared to the T2D-Veh group after two weeks of OA treatment despite matching of food intake, and this was maintained for 3-weeks post-treatment (Fig. 6*C*). Similar to the previous observation, T2D-OA mice were more glucose tolerant than T2D-Veh mice in the ipGTT performed two weeks after the removal of OA (Fig. 6, *D* and *E*).

### Glucose flux in muscle, fat and liver after OA treatment

**Figure 7:**
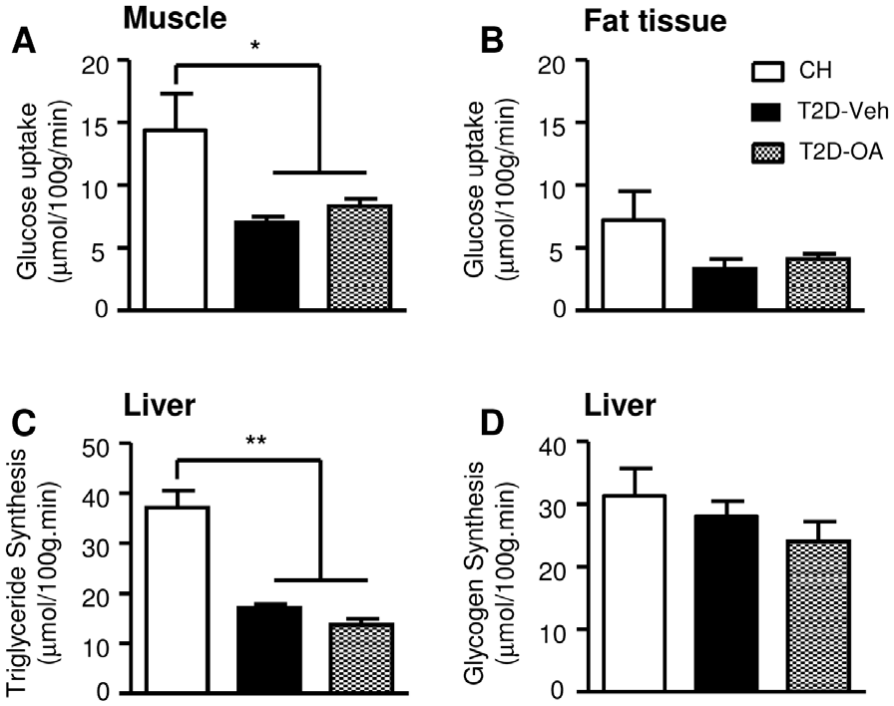
Changes in glucose flux into muscle, adipose tissue and liver 4 weeks after the removal of OA from the diet. An ipGTT (1 g/kg, ip) using 2-deoxy-D-[1,2-^3^H] glucose and D-[^14^C] glucose was performed 4 weeks after the cessation of OA treatment. At the end of the 40-min ipGTT, tissue samples were freeze-clamped immediately for the measurement of glucose uptake in quadriceps muscle (*A*) and epididymal fat (*B*), as well as the glucose incorporation into lipid (*C*) and glycogen (*D*) in the liver. * p<0.05, ** p<0.01 vs. CH, n = 6–12 per group.

To further investigate the mechanism underlying the sustained improvement in glycemia of OA treated T2D mice, we employed [^3^H]-2DG and [^14^C]-glucose tracers to measure the glucose uptake in muscle and adipose tissue during an ipGTT conducted 4 weeks after the cessation of OA treatment. As expected, T2D mice demonstrated reduced glucose uptake in muscle compared to CH-fed mice (Fig. 7*A*)(14.4±2.9 vs. 7.0±0.5 µmol/100 g/min, p<0.05). A similar tendency was evident in fat tissue (7.2±2.3 vs. 3.3±0.8 µmol/100 g/min, p>0.05; Fig. 7*B*). However, OA treatment of T2D mice did not cause any improvement of glucose uptake in these two tissues (Fig. 7*A, B*). Furthermore, measurement of glucose incorporation into glycogen and lipid in the liver (as a proxy of glucose influx) did not show any improvement in T2D-OA as compared to the T2D-Veh group (Fig. 7, *C* and *D*).

### Changes in key regulators of hepatic glucose production after OA treatment

**Figure 8:**
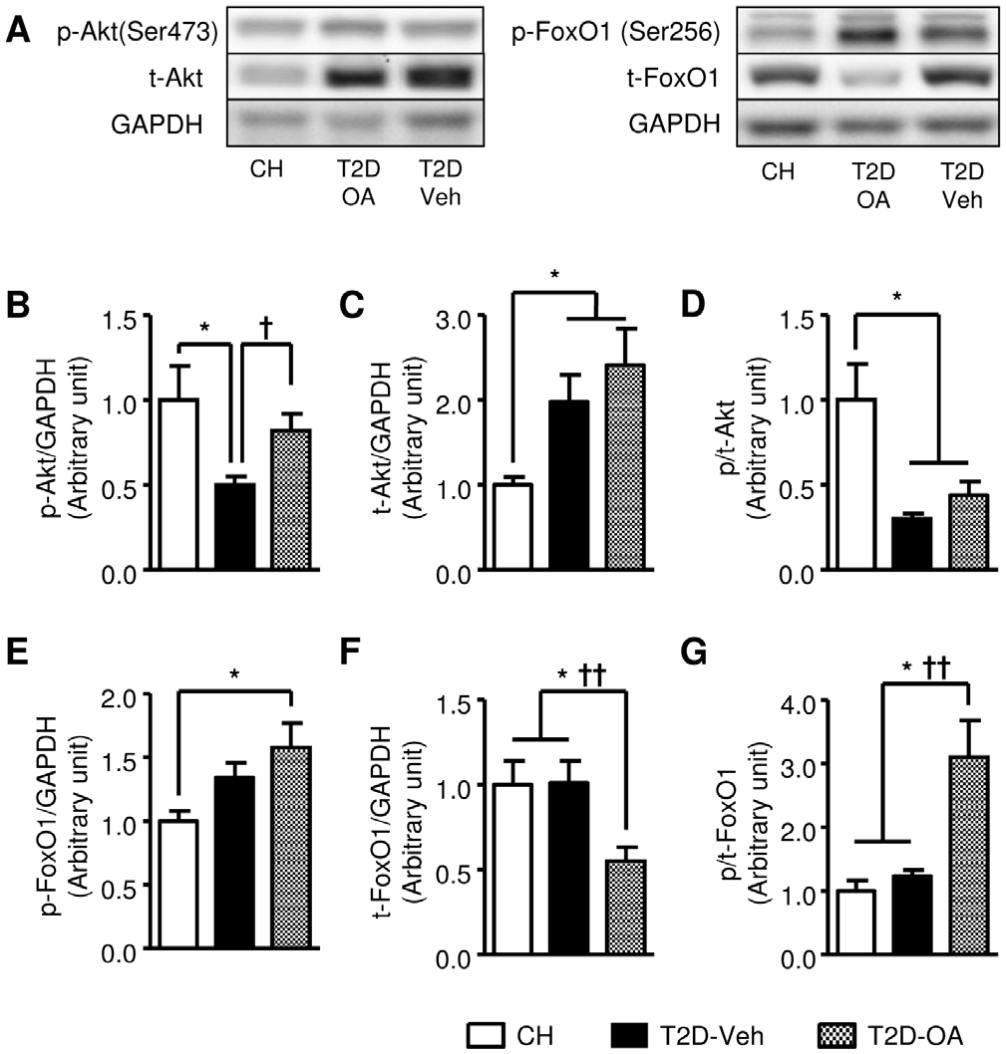
Changes in Akt and FoxO1 in the liver 4 weeks after the removal of OA. Four weeks after the cessation of OA treatment, mice were sacrificed following a 5–7 hour fast. Liver samples were freeze-clamped and stored at −80°C for subsequent Western blotting analysis. Representative Western blot images of phosphorylated and total Akt and FoxO1(*A*). Quantification of p-Akt/GAPDH (*B*), t-Akt/GAPDH (*C*), p-/t-Akt (*D*), p-FoxO1/GAPDH (*E*), t-FoxO1/GAPDH (*F*) and p-/t-FoxO1 (*G*). * p<0.05 vs. CH; † p<0.05, †† p<0.01 vs. T2D-Veh, n = 6–8 per group.

As hepatic glucose production is a major factor affecting glucose homeostasis [27], we investigated the key molecules (Akt and FoxO1 [28]) in the insulin signaling pathway that regulate hepatic gluconeogenesis and glucose production. As shown in Fig. 8*A–D*, compared to CH-fed mice, T2D mice displayed a 60% reduction of p-/t-Akt. The p-/t-Akt ratio was similar in T2D-OA and T2D-Veh mice. However, the absolute p-Akt level was lower in T2D-Veh mice compared to CH, but restored to CH levels in T2D-OA mice. In the case of FoxO1, T2D-OA mice demonstrated substantially increased p-/t-FoxO1 (200% increase) (p<0.01) and a 50% reduction in total FoxO1 protein compared to both T2D-Veh and CH mice (p<0.05) (Fig. 8, *A,E–G*).

**Figure 9:**
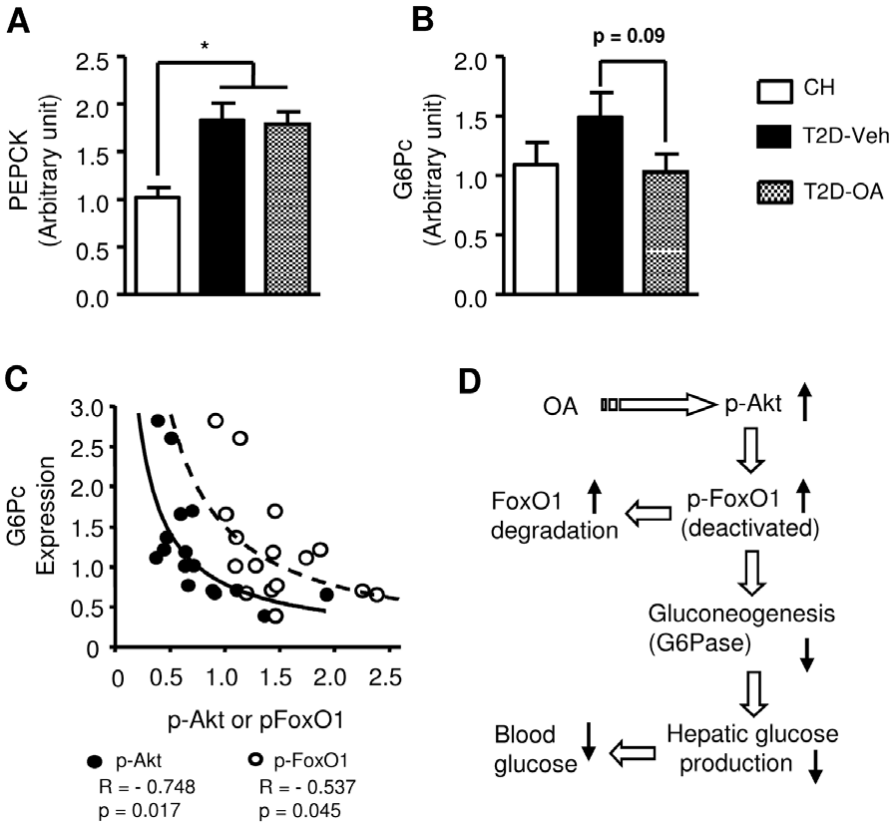
Changes in PEPCK and G6Pase expression in the liver 4 weeks after the removal of OA. Four weeks after the cessation of OA treatment, mice were sacrificed following a 5–7 hour fast. Liver samples were freeze-clamped and stored for subsequent analysis of PEPCK and G6Pase RNA expression. The expression levels of PEPCK and G6Pase mRNA relative to 18S (*A* and *B*). Correlation of p-Akt/GAPDH and FoxO1/GAPDH with G6Pase mRNA expression in T2D-Veh and T2D-OA groups by a best-fit regression analysis (*C*). * p<0.05 vs. CH, n = 6–8 per group. Proposed mechanism for the sustained reduction in hyperglycemia following the treatment of OA (*D*).

We next examined the transcriptional expression levels of the key enzymes controlling gluconeogenesis (PEPCK) and glucose production (G6Pase). As shown in Figure 9*A* and *B*, compared to CH-fed mice, T2D mice demonstrated an elevated expression of PEPCK (∼80% increase, p<0.05) and G6Pase (∼50% increase, p>0.05) at the transcriptional level. Interestingly, treatment of T2D mice with OA tended to reduce G6Pase RNA (∼30%, p = 0.09, Fig. 9*B*), while the level of PEPCK expression remained unchanged. Further analysis showed that there was a significant reversed correlation of both phosphorylated Akt(p<0.05) and phosphorylated FoxO1 (p<0.05) to the transcriptional expression level of G6Pase (Fig. 9*C*).

## Discussion

The present study investigated the chronic effect of OA, a triterpenoid selected from our recent screens [14], on hyperglycemia in diabetic mice and the contributing mechanisms involved. Our results show that OA did not affect hyperglycemia in T1D mice, but was able to reverse hyperglycemia in T2D mice, as predicted. Intriguingly, this reversed hyperglycemia was sustained well beyond the treatment period - for at least 4 weeks after cessation of OA treatment. Along with the correction of hyperglycemia, elevated urine glucose loss in T2D mice was also completely reversed and damage to renal structures was markedly attenuated. Although OA has been reported to lower hyperglycemia by counteracting insulin resistance within the period of its administration [29], [30], [31], to the best of our knowledge the sustained reversal of hyperglycemia for such a long duration after terminating the treatment has not been described before.

Since previous studies reported a hypoglycemic effect in insulin resistant HF-fed [29], [30], [31] and *db/db* mice [18], we first investigated the effects of OA on hyperglycemia in a T2D model generated by chronic HF feeding in combination with low doses of STZ. Chronic HF feeding in rodents is a widely used model of insulin resistance, associated with lipid accumulation in muscle and liver [7], [32]. However, HF feeding alone is insufficient to cause diabetes due to the capacity of pancreatic β-cells to increase insulin secretion in order to compensate for the insulin resistance [33]. Therefore, we administered multiple low doses of STZ to restrict the ability of pancreatic β-cells to increase insulin secretion, thereby generating hyperglycemia as previously reported [20], [21]. As expected, the combination of HF feeding and low doses of STZ resulted in marked hyperglycemia with the characteristics of overt type-2 diabetes, namely, failed compensatory increase in blood insulin levels with concurrent hypertriglyceridemia and fatty liver. Compared with CH-fed mice injected with STZ (T1D), HF-fed mice injected with STZ (T2D) showed significantly more severe hyperglycemia liver steatosis and visceral adiposity (Table 1).

Oral administration of OA to the T2D mouse model progressively reduced hyperglycemia to a level similar to that of normal mice within two weeks. Interestingly, after the termination of OA treatment the reversed hyperglycemia was retained for the remaining 4-week period of the study (Fig. 1*A*). Along with the reduced blood glucose level, T2D-OA mice also displayed an improved glucose tolerance at the end of the study when measured with the total area of glucose under the curve (Fig. 1*A, B*). While the hypoglycemic effect of an anti-diabetic compound is expected during the period of treatment, we are not aware of any other report showing similarly sustained correction of hyperglycemia after cessation of drug treatment. This dramatic improvement of glucose homeostasis for such a long time post-OA treatment is particularly interesting because sustained control of glycemia is an important target in the treatment of type-2 diabetes.

Possible mechanisms of the sustained reversal of hyperglycemia following the treatment of OA were investigated from several different perspectives. The half-life of OA in the circulation is reported to be less than 30 min in mice [34], suggesting that the reversal of hyperglycemia post-OA in the present study is unlikely to result from a prolonged presence of OA in the circulation. We therefore first investigated whether OA may induce sustained improvement of pancreatic β-cell function to increase insulin secretion, given that acute treatment of OA was previously reported to enhance insulin secretion in isolated rat β-cells [35]. Such a possibility has been implicated in several previous studies using a T1D model induced by STZ in CH-fed rodents [17], [19]. Conceivably, the sustained reversal of hyperglycemia may be achieved with a compensatory increase in insulin secretion as observed in the HF-fed mice in the present study (Table 1) or improved β-cell function as suggested for incretins [13]. However, our assessment showed that 2-week treatment of T2D mice with OA did not increase the number or enhance the function of β -cells (pancreatic insulin content or insulin secretion of β-cells in response to glucose stimulation). Furthermore, the blood insulin profiles during the ipGTT were not different between T2D mice with/without OA treatment. These results are also consistent with the inability of OA to lower hyperglycemia in STZ-injected CH-fed mice in the present study. The reason for the discrepancy of our results from the previous reports in this T1D rodent model [17], [19] is not clear. However, our findings in T2D and T1D models are internally consistent and collectively indicate that it is unlikely that OA exerts its beneficial effects on glycemia through an improvement in β-cell function.

It has been proposed that the inhibition of SGLT2 (a transporter for glucose re-absorption in the kidney) can increase glucose excretion in the urine and thereby reduce hyperglycemia for the treatment of type-2 diabetes [12], [13]. We therefore examined whether the sustained anti-hyperglycemic effect of OA was achieved by increasing urine glucose excretion. However, in contrast to an increase in the glucose excretion through the kidney, OA treatment significantly reduced the urine glucose level in T2D mice to the level of CH-fed mice. Furthermore, the diabetic nephropathy evident in T2D-Veh mice was abolished in T2D mice treated with OA, as indicated by increased tubular cell height, and decreased interstitial volume and glomerular turf area. These results rule out increased urine excretion of glucose as a mechanism for the reduced glucose level following OA treatment. These findings are consistent with a recent clinical trial which demonstrated the beneficial effects of an OA derivative for nephropathy in type-2 diabetes patients [9]. As OA can improve renal functions in diabetic mice by inhibiting the formation of advanced glycation endproducts [17], it is possible that the reversal of hyperglycemia following the treatment of OA contributed to the alleviated nephropathy.

The reduced food intake evident in T2D-OA mice during the period of OA treatment presented another potential mechanism underlying the sustained reduction in glycemia. Reduced food intake has been shown to have effects on fasting blood glucose levels, glucose tolerance and insulin sensitivity in mice and rats [36], [37]. However, it is unknown whether the on glycemia, can be maintained for days after food intake has normalised. To investigate possible influences of the different food intake pattern of T2D-Veh and T2D-OA groups, we conducted a pair-feeding study with an additional two groups of mice. Despite food intake of the pair-fed T2D-Veh and T2D-OA groups being matched throughout the study, T2D-Veh mice maintained significantly higher glycemia than T2D-OA mice from the second week of OA treatment and through to the end of the post-treatment period (Fig. 6*A–C*). Furthermore, pair-fed T2D-Veh mice were not as glucose tolerant as T2D-OA mice as evidenced by an ipGTT performed at the end of the study (Fig. 6*D, E*). These data indicate that the effect of OA to improve glucose homeostasis in diabetic mice does not rely on its effect to reduce food intake. In support of this interpretation, a recent study has demonstrated that an OA analogue can lower blood glucose in both HF-fed and db/db mice without affecting food intake [18].

Several studies have shown that triterpenoids, including OA, can reverse insulin resistance and glucose intolerance during the treatment period [18], [29], [30], [38]. The present study similarly observed that T2D-OA mice displayed significantly lower plasma glucose levels during ipGTT. Since OA treatment attenuates the glucose intolerance with similar blood insulin levels as T2D-Veh mice, the improved glucose tolerance and glycemia in T2D-OA mice is probably due to improved insulin sensitivity of the peripheral tissues.

Insulin resistance is closely related with increased accumulation of lipids in peripheral tissues [39] and a reduction in lipid content in these tissues is an effective means to improve insulin sensitivity [7]. The reversal of insulin resistance and glucose intolerance during OA treatment has been associated with a reduction of lipid accumulation in muscle and liver [18], [29], [30], [38]. As triterpenoids can acutely activate AMPK and promote fat oxidation [14], one plausible mechanism of the sustained efficacy to reduce hyperglycemia in the present study could conceivably be due to improved insulin action as a result of reduced lipid accumulation in muscle and liver. Such a mechanism has been demonstrated with berberine [40], and Abbot compound A [41] during the period of treatment. To investigate if OA improves insulin sensitivity through a similar mechanism, we measured the triglyceride levels in plasma and liver because a reduction in hepatic steatosis is able to normalize glycemia in type-2 diabetes [42]. Indeed, associated with the corrected glycemia, the triglyceride levels in plasma and liver were reduced to almost the normal levels of CH-mice during the period of OA administration. These results are similar to those recently reported in *db/db* mice within the treatment duration of an OA analogue, along with increased AMPK activity [18]. However, as the efficacy of OA on triglyceride levels in plasma and liver did not persist after cessation of OA treatment, the sustained reduction in glycemia is likely to involve alternative/additional mechanisms rather than the improvement in dyslipidemia and hepatic steatosis alone.

Glucose uptake into skeletal muscle and hepatic production of glucose are two major metabolic pathways responsible for glucose homeostasis. With the use of [^3^H]-2DG and D-[^14^C] glucose tracers, we demonstrated a reduction of glucose uptake into muscle and adipose tissue in T2D-Veh mice and there was no improvement of glucose uptake in these two tissues in T2D-OA mice. The lack of an effect of OA upon glucose uptake into muscle and liver indicated that the liver may be the major site for the sustained improvement of glycemia after the treatment with OA. To investigate the possible role of the liver, we first assessed glucose influx into this organ, but found no improvement in glucose incorporation into either glycogen or triglyceride (note that measurement of glucose uptake with 2DG in the liver is not valid). We next examined indicators of hepatic glucose production (efflux). Interestingly, we found that the insulin signaling transduction (indicated by the phosphorylation of Akt and FoxO1) regulating gluconeogenesis was significantly improved in OA treated mice. FoxO1 is a key transcription factor regulating hepatic gluconeogenesis. The phosphorylation of FoxO1 by Akt leads to its expulsion from the nucleus for degradation [28], [43]. A recent study has indicated that the Akt-FoxO1 signaling plays a key role in controlling the expression of G6Pase [43]. Indeed, we found a trend of reduced expression of G6Pase (p = 0.09). Importantly the suppressed expression of G6Pase was significantly correlated with increased phosphorylation of Akt and FoxO1 (both p<0.05). As G6Pase is the gate-keeping enzyme for hepatic glucose production, our data indicate that its down-regulation induced by the Akt/FoxO1 signaling pathway is a likely mechanism for the sustained improvement of glycemia after the treatment with OA (Fig. 9*E*).

In summary, the present study demonstrated a sustained effect of OA to reverse hyperglycemia in T2D mice induced by HF-feeding and STZ. Our data indicate that OA, a triterpenoid abundantly present in natural products, may be a potential drug for the sustained control of hyperglycemia in type-2 diabetes and related kidney complications independent of lipid metabolism, insulin secretion and glucose disappearance into muscle and fat tissue. Furthermore, our data suggest that the sustained improvement of glucose homeostasis is due, at least in part, to a suppression of gluconeogenesis in the liver mediated by the Akt/FoxO1 axis. The findings in our study also provide a proof of concept for the potential of triterpenoids as a promising source to explore new drugs for the long-term control or cure of hyperglycemia and diabetic kidney complications.
